# Supplementary material for: In-Situ Simulation for Enhancing Safety in Outpatient Hysteroscopy: Development and Evaluation of a Crisis Resource Management-Based Training Package
Source: MedEdPORTAL. 2026 Jun 5;22:11604. doi: 10.15766/mep_2374-8265.11604 (PMC13236966; doi:10.15766/mep_2374-8265.11604)
Supplement: Supplementary file 1 — Oversedation Case.docxHemorrhage Case.docxLAST Case.docxVasovagal Case.docxHemorrhaging Uterus Model.docxDebriefing Materials.docxCrisis Resource Management Primer.docxLatent Safety Threats Template.docxSelf-Efficacy Tool Presurvey.docxSelf-Efficacy Tool Postsurvey.docxParticipant Evaluation Form.docx [file mep_2374-8265.11604-s001.zip › mep_2374-8265.11604-s001/A. Oversedation Case.docx]

Appendix A. Oversedation Case

| **Appendix A: *MedEdPORTAL* Simulation Case Template**  **SIMULATION CASE TITLE:** Oversedation in the Outpatient Hysteroscopy Suite  **AUTHORS:** Chelsie Warshafsky and Adam Garber  **LEARNER AUDIENCE:** Physicians and nurses | |
| --- | --- |
| **PATIENT NAME:** Ivy Tumuch  **PATIENT AGE:** 47  **CHIEF COMPLAINT:** Drowsiness  **PHYSICAL SETTING:** Outpatient hysteroscopy suite | |
|  | |
| **Brief Narrative Description of Case** | Ivy Tumuch is a 47yo G3P3 presenting to the outpatient hysteroscopy suite for a global endometrial ablation for heavy menstrual bleeding and adenomyosis. She has a history of anxiety, is otherwise healthy, and has no known allergies. In this case the patient is extremely anxious, so the nurses give IV sedation as soon as the safety checklist is done. Soon after the patient becomes drowsy and then unresponsive. |
| **Primary Learning Objectives** | 1. Recognize the presenting signs and symptoms of oversedation and implement treatment. 2. Initiate management of oversedation in an outpatient surgical suite and recognize the specific antidotes required based on medications given. 3. Appraise existing equipment and unit protocols; identify latent safety threats in response to oversedation in the outpatient hysteroscopy setting. 4. Apply the principles of crisis resource management with a focus on role allocation and task prioritization. |
| **Critical Actions** | - Identify signs and symptoms of oversedation - Manage oversedation appropriately - Recognize when to call for help - Utilize resources available in the specific outpatient setting - Identify appropriate disposition for patient - Demonstrate crisis resource management skills |
| **Learner Preparation or Prework** | - Inform participants that simulation is a safe environment solely for practice and learning purposes - Learners will be working as a team - Orient learners to the mannequin, monitors, and equipment - Encourage learners to act as they would in a real-life scenario getting equipment, giving medications, speaking to the patient, etc. - Explain the roles of each participant - Explain that facilitator will be the voice of the patient - Explain that the facilitator will communicate any pertinent information the learners need on request |

| Initial Presentation | | | |
| --- | --- | --- | --- |
| **Initial Vital Signs** | Alert and oriented  HR 90, BP 120/70, RR 16, SaO2t 98% | | |
| **Overall Setting and Appearance** | Mannequin set up in the hysteroscopy suite in dorsal lithotomy with legs in stirrups ready for procedure to begin. | | |
| **Standardized Participants (and Their Roles in the Room at Case Start)** | Prior to the start of the simulation, the facilitator and simulation technician will orient the learners to the mannequin, monitors and other equipment. If performed in-situ, facilitators will stand at the back of the room. If in a simulation centre setting, facilitators will go behind a one-way mirror.  The facilitator will assign roles to the participants: scrub nurse and physician. If available in the specific setting can also assign a circulating nurse and/or medical learner.  Learners will evaluate the patient together on initial presentation.  The facilitator will be the voice of the patient and will provide history (on inquiry) and physical exam findings, and provide any information requested.  The facilitator will guide learners through timepoints. The simulation technician will then change vital signs accordingly. | | |
| **HPI** | Facilitator will provide the following introduction:  *“*Ivy Tumuch is a 47yo G3P3 presenting to the outpatient hysteroscopy suite for a global endometrial ablation for heavy menstrual bleeding and adenomyosis. She has a history of anxiety, is otherwise healthy, and has no known allergies. She is waiting for you to begin the procedure.”  Learners will obtain further complete history. History must include questions about previous medications taken, at which time they will be informed that the patient took anxiolytics prior to the procedure. | | |
| **Past Medical/Surgical History** | **Past Obstetrical History** | **Medications** | **Allergies** |
| Anxiety  Heavy menstrual bleeding  Adenomyosis | Three previous uncomplicated spontaneous vaginal deliveries | Ativan | NKDA |
| **Physical Examination** | | | |
| **General** | No apparent distress | | |
| **HEENT** |  | | |
| **Neck** |  | | |
| **Lungs** | Clear to auscultation bilateral | | |
| **Cardiovascular** | Normal S1 S2, regular rate/rhythm | | |
| **Abdomen** | Soft, nontender, nondistended, no rebound or guarding | | |
| **Neurological** | Alert and oriented | | |
| **Skin** |  | | |
| **GU** | Normal vulva, vagina, cervix, anteverted globular uterus | | |
| **Psychiatric** | Very anxious | | |

| Instructor Notes - Changes and CASE Branch Points | | | | | |
| --- | --- | --- | --- | --- | --- |
| **State** | **Patient Status** | **Facilitator**  *(Patient simulator)* | **Learner Actions** | **Trigger**  *(Action causing state to change)* | **Teaching Points** |
| **Baseline**  (0-5 min) | Alert and oriented   - HR- 90 - BP- 120/70 - RR- 16 - SaO2- 98% | - Responds to questions appropriately | - Complete safety checklist - Nurse gives IV sedation-   - Fentanyl 50-100 mcg   - Midazolam 1-2mg | - IV sedation given | - Safety checklist - Medication dosing |
| **Early signs**  (5-7 min) | Becomes drowsy   - HR- 90 - BP- 120/70 - RR- 12 - SaO2- 93% | - Complains of drowsiness - Slurs speech - Discloses taking Ativan before coming | - Recognize early signs of oversedation | - Time- should last 1-2 minutes | - Drowsiness is an early symptom of oversedation and should prompt further clinical assessment |
| **Decreased level of consciousness**  (7-10 mins) | Unresponsive, slow breathing   - HR- 70 - BP- 98/62 - RR- 8 - SaO2- 85% | - Do not respond verbally to stimulus - Decrease O2sat over 1 minute | - Recognize change in clinical status - Apply supplemental oxygen - Call for help - Call for crash cart | - Time- should last 3-4 minutes | - Oversedation can progress to decreased level of consciousness and respiratory arrest - Consider human resource response on unit |
| **Respiratory Arrest**  (10-15 mins) | Respiratory arrest   - HR- 70 - BP- 80/50 - RR- 0 - SaO2- 70% |  | - Diagnose oversedation - Start bag ventilation - Call Code Blue/ Rapid Response Team (if available) - Initiate treatment with antidotes | - Bag ventilation will lead to improved O2sat - Providing antidotes will lead to return of respiratory effort - If no antidote given, end scenario after 5 minutes of bag ventilation | - Consider differential diagnosis including oversedation - Treatment of oversedation includes antidotes- consider availability on unit |
| **Resolution**  (>15 mins)  *Only if antidote given* | Return of respiratory effort   - HR- 110 - BP- 120/75 - RR- 12 - SaO2- 93% | - Return vital signs | - Recognize return of respiratory effort - Provide supportive care - Discuss disposition planning | - End scenario when disposition discussion is completed | - Continue supportive care - Review local protocols for urgent hospital/ICU transfer |

min- minutes; HR- heart rate; BP- blood pressure; RR- respiratory rate; SaO2- oxygen saturation; mcg- micrograms; mg- milligrams.

**Ideal Scenario Flow**

The learners enter the room to find the patient ready for her procedure. They complete a pre-operative safety checklist, and the nurse then begins to administer IV sedation. The patient begins to become drowsy with slurred speech. Upon questioning the team discovers the patient took Ativan prior to the procedure due to anxiety. They apply supplemental oxygen and call for help as the patient becomes unresponsive. The team recognizes oversedation and treat with the appropriate antidote medications. Respiratory effort returns and supportive care is provided. The team discusses disposition planning and determines the best setting for the patient.
