# Supplementary material for: In-Situ Simulation for Enhancing Safety in Outpatient Hysteroscopy: Development and Evaluation of a Crisis Resource Management-Based Training Package
Source: MedEdPORTAL. 2026 Jun 5;22:11604. doi: 10.15766/mep_2374-8265.11604 (PMC13236966; doi:10.15766/mep_2374-8265.11604)
Supplement: Supplementary file 1 — Oversedation Case.docxHemorrhage Case.docxLAST Case.docxVasovagal Case.docxHemorrhaging Uterus Model.docxDebriefing Materials.docxCrisis Resource Management Primer.docxLatent Safety Threats Template.docxSelf-Efficacy Tool Presurvey.docxSelf-Efficacy Tool Postsurvey.docxParticipant Evaluation Form.docx [file mep_2374-8265.11604-s001.zip › mep_2374-8265.11604-s001/B. Hemorrhage Case.docx]

Appendix B. Hemorrhage Case

| **Appendix A: *MedEdPORTAL* Simulation Case Template**  **SIMULATION CASE TITLE:** Hemorrhage in the Outpatient Hysteroscopy Suite  **AUTHORS:** Chelsie Warshafsky and Adam Garber  **LEARNER AUDIENCE:** Physicians and nurses | |
| --- | --- |
| **PATIENT NAME:** Hema Rajakumar  **PATIENT AGE:** 28  **CHIEF COMPLAINT:** Bleeding  **PHYSICAL SETTING:** Outpatient hysteroscopy suite | |
|  | |
| **Brief Narrative Description of Case** | Hema Rajakumar is a 28yo G1 who underwent a termination of pregnancy at 11w3d at an outpatient facility 2 weeks ago. She had a repeat ultrasound due to persistent bleeding, which revealed a retained product of conception with vascular flow. She presenting to the outpatient hysteroscopy suite for a hysteroscopic resection of retained products of conception. The patient receives a paracervical block. Once the dilation is started a significant amount of bleeding is encountered. The team must recognize this, apply monitors, temporizing measures, and transfer the patient for further management. |
| **Primary Learning Objectives** | 1. Recognize the presenting signs and symptoms of hemorrhage and implement treatment. 2. Initiate management of hemorrhage in an outpatient surgical suite and recognize the specific antidotes required based on medications given. 3. Appraise existing equipment and unit protocols; identify latent safety threats in response to hemorrhage in the outpatient hysteroscopy setting. 4. Apply the principles of crisis resource management with a focus on resource allocation and active followership. |
| **Critical Actions** | - Identify signs and symptoms of hemorrhage - Manage hemorrhage appropriately - Recognize when to call for help - Utilize resources available in the specific outpatient setting - Identify appropriate disposition for patient - Demonstrate crisis resource management skills |
| **Learner Preparation or Prework** | - Inform participants that simulation is a safe environment solely for practice and learning purposes - Learners will be working as a team - Orient learners to the mannequin, monitors, and equipment - Encourage learners to act as they would in a real-life scenario getting equipment, giving medications, speaking to the patient, etc. - Explain the roles of each participant - Explain that facilitator will be the voice of the patient - Explain that the facilitator will communicate any pertinent information the learners need on request |

| Initial Presentation | | | |
| --- | --- | --- | --- |
| **Initial Vital Signs** | Alert and oriented  No monitors on | | |
| **Overall Setting and Appearance** | Mannequin set up in the hysteroscopy suite in dorsal lithotomy with legs in stirrups ready for procedure to begin. | | |
| **Standardized Participants (and Their Roles in the Room at Case Start)** | Prior to the start of the simulation, the facilitator and simulation technician will orient the learners to the mannequin, monitors and other equipment. If performed in-situ, facilitators will stand at the back of the room. If in a simulation centre setting, facilitators will go behind a one-way mirror.  The facilitator will assign roles to the participants: scrub nurse and physician. If available in the specific setting can also assign a circulating nurse and/or medical learner.  Learners will evaluate the patient together on initial presentation.  The facilitator will be the voice of the patient and will provide history (on inquiry) and physical exam findings, and provide any information requested.  The facilitator will guide learners through timepoints. The simulation technician will then change vital signs accordingly. | | |
| **HPI** | Facilitator will provide the following introduction:  “Hema Rajakumar is a 28yo G1 who underwent a termination of pregnancy at 11w3d at an outpatient facility 2 weeks ago. She had a repeat ultrasound due to persistent bleeding, which revealed a 22x25mm retained product of conception with vascular flow. She presenting to the outpatient hysteroscopy suite for a hysteroscopic resection of retained products of conception. She is otherwise healthy, smokes 1g of cannabis daily, and is allergic to penicillin. She is waiting for you to begin the procedure.”  The patient receives a paracervical block in the usual fashion, which causes the expected tachycardia secondary to epinephrine. Once the dilation is started a significant amount of bleeding is encountered. The team must recognize this, apply monitors, temporizing measures, and transfer the patient for further management. | | |
| **Past Medical/Surgical History** | **Past Obstetrical History** | **Medications** | **Allergies** |
| Healthy  Suction dilation and curettage- 2 weeks ago | G1- recent TA | None | Penicillin |
| **Physical Examination** | | | |
| **General** | No apparent distress | | |
| **HEENT** |  | | |
| **Neck** |  | | |
| **Lungs** | Clear to auscultation bilateral | | |
| **Cardiovascular** | Normal S1 S2, regular rate/rhythm | | |
| **Abdomen** | Soft, nontender, nondistended, no rebound or guarding | | |
| **Neurological** | Alert and oriented | | |
| **Skin** |  | | |
| **GU** | Normal vulva, vagina, cervix, anteverted uterus | | |
| **Psychiatric** |  | | |
| **Transvaginal Ultrasound:** Anteverted uterus. Heterogeneous myometrium. 22x25mm retained product of conception with vascular flow. Normal adnexa bilaterally. | | | |

| Instructor Notes - Changes and CASE Branch Points | | | | | |
| --- | --- | --- | --- | --- | --- |
| **State** | **Patient Status** | **Facilitator**  *(Patient Simulator)* | **Learner Actions** | **Trigger**  *(Action causing state to change)* | **Teaching Points** |
| **Baseline**  (0-5 min) | Alert and oriented | - Responds to questions appropriately | - Complete safety checklist - MD gives paracervical block | - Paracervical block given | - Safety checklist |
| **Early signs**  (5-7 min) | Alert and oriented | - *Opens IV tubing to allow blood to flow into uterus* | - Starts procedure *(Note: bleeding can start with dilation or resection depending on model available)* - Recognizes large volume of bleeding - Apply monitors | - Significant bleeding | - Recognize high volume of bleeding and anticipate worsening clinical status |
| **Acute Hemorrhage**  (7-10 mins) | Alert and anxious   - HR- 105 - BP- 110/70 - RR- 14 - SaO2- 98% | - Complains of feeling lightheaded | - Recognize change in clinical status - Insert 2 large bore IVs and start fluids - Call for help - Lower head of bed - Ask for Foley - Ask for TXA, uterotonics | - EBL: 1L (can be announced if not clear based on model used) | - Initial management of acute hemorrhage - Consider human resource response on unit |
| **Ongoing Hemorrhage**  (10-15 mins) | Feeling unwell   - HR- 130 - BP- 85/55 - RR- 20 - SaO2- 93% | - Asks if everything is okay - Complains of nausea | - Insert intrauterine Foley until resistance - Give uterotonics | - Foley insertion - Fluid resuscitation - If not done, progress after 5 minutes (EBL: 2L) | - Review temporizing measures for hemorrhage - Evaluate equipment availability on unit |
| **Resolution**  (>15 mins) | Stabilization   - HR- 100 - BP- 120/75 - RR- 12 - SaO2- 95% | - Improved vital signs - Decreased bleeding | - Recognize stability - Arrange transfer to ED - Handover to EMS/receiving physician | - End scenario when handover discussion is completed | - Continue supportive care - Review local protocols for urgent hospital/ICU transfer |

HR- heart rate; BP- blood pressure; RR- respiratory rate; SaO2- oxygen saturation; EBL- estimated blood loss; L- litres.

**Ideal Scenario Flow**

The learners enter the room to find the patient ready for her procedure. They complete a pre-operative safety checklist. The physician performs a paracervical block and begins to dilate the cervix (or insert hysteroscope, depending on model available). Significant bleeding begins due to disruption of the vascular retained product of conception. The team begins resuscitative measures, provide mechanical compression and medical management. The patient is stabilized, and the team arranges for transfer to the ED for further management in hospital.
