# Supplementary material for: In-Situ Simulation for Enhancing Safety in Outpatient Hysteroscopy: Development and Evaluation of a Crisis Resource Management-Based Training Package
Source: MedEdPORTAL. 2026 Jun 5;22:11604. doi: 10.15766/mep_2374-8265.11604 (PMC13236966; doi:10.15766/mep_2374-8265.11604)
Supplement: Supplementary file 1 — Oversedation Case.docxHemorrhage Case.docxLAST Case.docxVasovagal Case.docxHemorrhaging Uterus Model.docxDebriefing Materials.docxCrisis Resource Management Primer.docxLatent Safety Threats Template.docxSelf-Efficacy Tool Presurvey.docxSelf-Efficacy Tool Postsurvey.docxParticipant Evaluation Form.docx [file mep_2374-8265.11604-s001.zip › mep_2374-8265.11604-s001/C. LAST Case.docx]

Appendix C. Local Anesthetic Systemic Toxicity (LAST) Case

| **Appendix A: *MedEdPORTAL* Simulation Case Template**  **SIMULATION CASE TITLE:** Local Anesthetic Systemic Toxicity (LAST) in the Outpatient Hysteroscopy Suite  **AUTHORS:**  Chelsie Warshafsky and Adam Garber  **LEARNER AUDIENCE:** Physicians and nurses | |
| --- | --- |
| **PATIENT NAME:** Shiloh Cane  **PATIENT AGE:** 36  **CHIEF COMPLAINT:** Perioral numbness and dizziness  **PHYSICAL SETTING:** Outpatient hysteroscopy suite | |
|  | |
| **Brief Narrative Description of Case** | Shiloh Cane is a 36yo G0 presenting to the outpatient hysteroscopy suite for a hysteroscopic polypectomy discovered on fertility workup. A saline infusion sonohysterogram showed a 12x8mm fundal polyp. She is otherwise healthy, takes prenatal vitamins, and has no known allergies. The physician performs a paracervical block, and the patient complains of perioral numbness, dizziness, and tinnitus, then becomes unresponsive. |
| **Primary Learning Objectives** | 1. Recognize the presenting signs and symptoms of LAST and implement treatment. 2. Initiate management of cardiac arrest in an outpatient surgical suite and recognize the specific considerations that differ from standard ACLS protocols. 3. Appraise existing equipment and unit protocols; identify latent safety threats in response to LAST in the outpatient hysteroscopy setting. 4. Apply the principles of crisis resource management with a focus on establishing leadership and practicing closed-loop communication. |
| **Critical Actions** | - Identify signs and symptoms of LAST - Initiate ACLS management appropriately - Acknowledge the differences in ACLS protocols in patients with LAST - Recognize when to call for help - Utilize resources available in the specific outpatient setting - Identify appropriate disposition for patient - Demonstrate crisis resource management skills |
| **Learner Preparation or Prework** | - Inform participants that simulation is a safe environment solely for practice and learning purposes - Learners will be working as a team - Orient learners to the mannequin, monitors, and equipment - Encourage learners to act as they would in a real-life scenario getting equipment, giving medications, speaking to the patient, etc. - Explain the roles of each participant - Explain that facilitator will be the voice of the patient - Explain that the facilitator will communicate any pertinent information the learners need on request |

| Initial Presentation | | | |
| --- | --- | --- | --- |
| **Initial Vital Signs** | Alert and oriented  No monitors on | | |
| **Overall Setting and Appearance** | Mannequin set up in the hysteroscopy suite in dorsal lithotomy with legs in stirrups ready for procedure to begin. | | |
| **Standardized Participants (and Their Roles in the Room at Case Start)** | Prior to the start of the simulation, the facilitator and simulation technician will orient the learners to the mannequin, monitors and other equipment. If performed in-situ, facilitators will stand at the back of the room. If in a simulation centre setting, facilitators will go behind a one-way mirror.  The facilitator will assign roles to the participants: scrub nurse and physician. If available in the specific setting can also assign a circulating nurse and/or medical learner.  Learners will evaluate the patient together on initial presentation.  The facilitator will be the voice of the patient and will provide history (on inquiry) and physical exam findings, and provide any information requested.  The facilitator will guide learners through timepoints. The simulation technician will then change vital signs accordingly. | | |
| **HPI** | Facilitator will provide the following introduction:  “Shiloh Cane is a 36yo G0 presenting to the outpatient hysteroscopy suite for a hysteroscopic polypectomy discovered on fertility workup. A saline infusion sonohysterogram showed a 12x8mm fundal polyp. She is otherwise healthy, takes prenatal vitamins, and has no known allergies. She is waiting for you to begin the procedure.” | | |
| **Past Medical/Surgical History** | **Past Obstetrical History** | **Medications** | **Allergies** |
| Healthy | G0 | Prenatal vitamins | NKDA |
| **Physical Examination** | | | |
| **General** | No apparent distress | | |
| **HEENT** |  | | |
| **Neck** |  | | |
| **Lungs** | Clear to auscultation bilateral | | |
| **Cardiovascular** | Normal S1 S2, regular rate/rhythm | | |
| **Abdomen** | Soft, nontender, nondistended, no rebound or guarding | | |
| **Neurological** | Alert and oriented | | |
| **Skin** |  | | |
| **GU** | Normal vulva, vagina, cervix, anteverted uterus | | |
| **Psychiatric** |  | | |
| Saline infusion sonohysterogram: Normal anteverted uterus. Endometrial thickness 2mm anterior, 2mm posterior. 12x8mm fundal polyp. | | | |

| Instructor Notes - Changes and CASE Branch Points | | | | | |
| --- | --- | --- | --- | --- | --- |
| **State** | **Patient Status** | **Facilitator**  *(Patient simulator)* | **Learner Actions** | **Trigger**  *(Action causing state to change)* | **Teaching Points** |
| **Baseline**  (0-5 min) | Alert and oriented | - Responds to questions appropriately | - Complete safety checklist - Gyne gives paracervical block | - Paracervical block given | - Safety checklist |
| **Early signs**  (5-7 min) | Complains of:   - Palpitations - Tinnitus - Perioral numbness | - Complains of tingling around mouth | - Recognize early signs of LAST - Apply monitors | - Time- should last 1-2 minutes | - Perioral numbness and tinnitus are early symptoms of LAST and should prompt further clinical assessment |
| **Decreased level of consciousness**  (7-10 mins) | Unresponsive, still breathing   - HR- 105 - BP- 130/85 - RR- 8 - SaO2- 85% | - Do not respond verbally to stimulus - Decrease SaO2 over 1 minute | - Recognize change in clinical status - Apply supplemental oxygen - Call for help - Call for crash cart | - Time- should last 3-4 minutes | - LAST can progress to decreased level of consciousness and coma - Consider human resource response on unit |
| **Cardiac Arrest**  (10-15 mins) | Asystolic arrest   - HR- 0 - BP- undetectable - RR- 0 - SaO2- 60% | If asked directly can confirm lipid emulsion is available and provide supplies | - Diagnose cardiac arrest and start ACLS - Call Code Blue/Rapid Response Team (if available) - Initiate treatment with lipid emulsion | - Lipid emulsion started - If no lipids given, progress after 5 minutes of ACLS | - Consider differential diagnosis including LAST - Treatment of LAST includes IV lipid emulsion- consider availability on unit - ACLS protocols differ when LAST is suspected |
| **Resolution**  (>15 mins) | Return of spontaneous circulation   - HR- 100 - BP- 120/75 - RR- 12 - SaO2- 93% | - Return vital signs | - Recognize ROSC - Provide supportive care - Discuss disposition planning | - End scenario when disposition discussion is completed | - Continue supportive care and lipid emulsion - Review local protocols for urgent hospital/ICU transfer |

HR- heart rate; BP- blood pressure; RR- respiratory rate; SaO2- oxygen saturation; LAST- local anesthetic systemic toxicity; ACLS- advanced cardiac life support; ROSC- return of spontaneous circulation.

**Ideal Scenario Flow**

The learners enter the room to find the patient ready for her procedure. They complete a pre-operative safety checklist. The physician performs a paracervical block and the patient complains of early signs of local anesthetic toxicity, then decompensates and becomes unresponsive. The team should recognize the change in clinical status, begin ACLS, and initiate lipid emulsion treatment. The patient is stabilized, and the team arranges for transfer to the ED/ICU for further management in hospital.x
