# Supplementary material for: In-Situ Simulation for Enhancing Safety in Outpatient Hysteroscopy: Development and Evaluation of a Crisis Resource Management-Based Training Package
Source: MedEdPORTAL. 2026 Jun 5;22:11604. doi: 10.15766/mep_2374-8265.11604 (PMC13236966; doi:10.15766/mep_2374-8265.11604)
Supplement: Supplementary file 1 — Oversedation Case.docxHemorrhage Case.docxLAST Case.docxVasovagal Case.docxHemorrhaging Uterus Model.docxDebriefing Materials.docxCrisis Resource Management Primer.docxLatent Safety Threats Template.docxSelf-Efficacy Tool Presurvey.docxSelf-Efficacy Tool Postsurvey.docxParticipant Evaluation Form.docx [file mep_2374-8265.11604-s001.zip › mep_2374-8265.11604-s001/D. Vasovagal Case.docx]

Appendix D. Vasovagal Case

| **Appendix A: *MedEdPORTAL* Simulation Case Template**  **SIMULATION CASE TITLE:** Vasovagal Episode on the Outpatient Hysteroscopy Suite  **AUTHORS:**  Chelsie Warshafsky and Adam Garber  **LEARNER AUDIENCE:** Physicians and nurses | |
| --- | --- |
| **PATIENT NAME:** Anita Trendelenburg  **PATIENT AGE:** 31  **CHIEF COMPLAINT:** Vasovagal episode  **PHYSICAL SETTING:** Outpatient hysteroscopy suite | |
|  | |
| **Brief Narrative Description of Case** | Anita Trendelenburg is a 31yo G3A3 who underwent a hysteroscopic lysis of adhesions for treatment of secondary infertility. Her history is significant for three therapeutic abortions, a LEEP procedure, and anxiety. She received a paracervical block and IV sedation. The cervix was stenotic, and it was difficult to access the cavity. Once this was achieved the procedure went well. While walking back to the recovery room she experiences a vasovagal episode in the hallway and becomes transiently unresponsive. |
| **Primary Learning Objectives** | 1. Consider differential diagnosis of acute hypotension following an outpatient hysteroscopy procedure. 2. Recognize signs and symptoms of a vasovagal episode and initiate management. 3. Appraise existing equipment and recovery room protocols following outpatient hysteroscopy procedures; identify latent safety threats. 4. Apply the principles of crisis resource management, with a focus on situational awareness and anticipating next steps. |
| **Critical Actions** | - Identify signs and symptoms of a vasovagal episode - Initiate supportive care appropriately - Determine how to best care for a patient in unfamiliar environments i.e. hallway - Utilize resources available in the specific outpatient setting - Identify appropriate disposition for patient - Demonstrate crisis resource management skills |
| **Learner Preparation or Prework** | - Inform participants that simulation is a safe environment solely for practice and learning purposes - Learners will be working as a team - Orient learners to the mannequin, monitors, and equipment - Encourage learners to act as they would in a real-life scenario getting equipment, giving medications, speaking to the patient, etc. - Explain the roles of each participant - Explain that facilitator will be the voice of the patient - Explain that the facilitator will communicate any pertinent information the learners need on request |

| Initial Presentation | | | |
| --- | --- | --- | --- |
| **Initial Vital Signs** | Alert and oriented  No monitors on | | |
| **Overall Setting and Appearance** | Patient is in the hysteroscopy suite and ready to be transferred back to the recovery area as her procedure is completed. | | |
| **Standardized Participants (and Their Roles in the Room at Case Start)** | Prior to the start of the simulation, the facilitator and simulation technician will orient the learners to the patient actor, monitors and other equipment. If performed in-situ, facilitators will stand at the back of the room. If in a simulation centre setting, facilitators will go behind a one-way mirror.  The facilitator will assign roles to the participants: scrub nurse and physician. If available in the specific setting can also assign a circulating nurse and/or medical learner.  Learners will evaluate the patient together on initial presentation.  The facilitator will be the voice of the patient and will provide history (on inquiry) and physical exam findings, and provide any information requested.  The facilitator will guide learners through timepoints. The simulation technician will then change vital signs accordingly. | | |
| **HPI** | Facilitator will provide the following introduction:  “Anita Trendelenburg is a 31yo G3A3 who underwent a hysteroscopic lysis of adhesions for treatment of secondary infertility. Her history is significant for three therapeutic abortions, a LEEP procedure, and anxiety. She received a paracervical block and IV sedation. The cervix was stenotic, and it was difficult to access the cavity. Once this was achieved the procedure went well. She is about to be transferred to the recovery room.” | | |
| **Past Medical/Surgical History** | **Past Obstetrical History** | **Medications** | **Allergies** |
| Anxiety  LEEP  D&C x3 | G3A3 | None | NKDA |
| **Physical Examination** | | | |
| **General** | No apparent distress | | |
| **HEENT** |  | | |
| **Neck** |  | | |
| **Lungs** | Clear to auscultation bilateral | | |
| **Cardiovascular** | Normal S1 S2, regular rate/rhythm | | |
| **Abdomen** | Soft, mild cramping post-procedure | | |
| **Neurological** | Alert and oriented | | |
| **Skin** |  | | |
| **GU** | Normal vulva, vagina, stenotic cervix, anteverted uterus | | |
| **Psychiatric** |  | | |

| Instructor Notes - Changes and CASE Branch Points | | | | | |
| --- | --- | --- | --- | --- | --- |
| **State** | **Patient Status** | **Facilitator**  *(Patient Simulator)* | **Learner Actions** | **Trigger**  *(Action causing state to change)* | **Teaching Points** |
| **Baseline**  (0-5 min) | Alert and oriented   - HR- 75 - BP- 110/70 - RR- 14 - SaO2- 98% | - Starts walking from procedure room to recovery room. | - Nurse is attending to patient and assesses vitals | - Patient reports feeling flushed |  |
| **Vasovagal**  (5-7 min) | Feels unwell then becomes unresponsive   - HR- 50 - BP- 75/45 - RR- 12 - SaO2- 94% | - Patient reports feeling hot and losing vision - Falls to ground - Becomes unresponsive | - Nurse calls physician for help - Repeat vitals | - Unresponsive x2 minutes | - Recognize acute hypotensive episode |
| **Management**  (7-10 mins) | Becomes responsive   - HR- 65 - BP- 85/60 - RR- 12 - SaO2- 94% | - Confused - Patient feels nauseated | - ABCs - 2 large bore IVs - Elevate legs - Review differential diagnosis - Call for help | - Review differential diagnosis | - Initial management of acute hypotension - Consider human resource response on unit |
| **Resolution**  (>15 mins) | Stabilization   - HR- 75 - BP- 120/75 - RR- 12 - SaO2- 97% | - Increasingly alert | - Recognize stability - Recognize likely cause- vasovagal episode - Continue supportive care - Disposition discussion | - End scenario when disposition discussion is completed | - Continue supportive care - Review local protocols for postoperative monitoring in outpatient suite |

HR- heart rate; BP- blood pressure; RR- respiratory rate; SaO2- oxygen saturation; ABCs- airway breathing circulation.

**Ideal Scenario Flow**

The nurse is present with the patient to start and walks her out of the procedure suite. The patient has a vasovagal episode in the hallway, falls to the ground and is unresponsive briefly. The nurse must recognize the clinical situation, call the physician for help, and start supportive care while in the hallway. The patient is stabilized, moved to the recovery area, and a disposition plan is made.
