# Supplementary material for: In-Situ Simulation for Enhancing Safety in Outpatient Hysteroscopy: Development and Evaluation of a Crisis Resource Management-Based Training Package
Source: MedEdPORTAL. 2026 Jun 5;22:11604. doi: 10.15766/mep_2374-8265.11604 (PMC13236966; doi:10.15766/mep_2374-8265.11604)
Supplement: Supplementary file 1 — Oversedation Case.docxHemorrhage Case.docxLAST Case.docxVasovagal Case.docxHemorrhaging Uterus Model.docxDebriefing Materials.docxCrisis Resource Management Primer.docxLatent Safety Threats Template.docxSelf-Efficacy Tool Presurvey.docxSelf-Efficacy Tool Postsurvey.docxParticipant Evaluation Form.docx [file mep_2374-8265.11604-s001.zip › mep_2374-8265.11604-s001/E. Hemorrhaging Uterus Model.docx]

**Appendix E. Hemorrhaging Uterus Model**

Steps:

1. Obtain model of female pelvis with built-in uterus or obtain rubber model of uterus with patent cervix.
2. Cut small hole on fundus of uterus.
3. Insert IV tubing through hole in uterus and secure.
4. Place inside existing female pelvis model if uterus not already built in.
5. Connect fake blood (bag of water with red food colouring) inside pressure bag to IV tubing to be opened by facilitators when bleeding should begin.

Low fidelity alternatives:

1. Create uterus out of foam pool noodle.
2. Indicate bleeding with red fabric or cards.
3. Verbally tell participants there is bleeding.


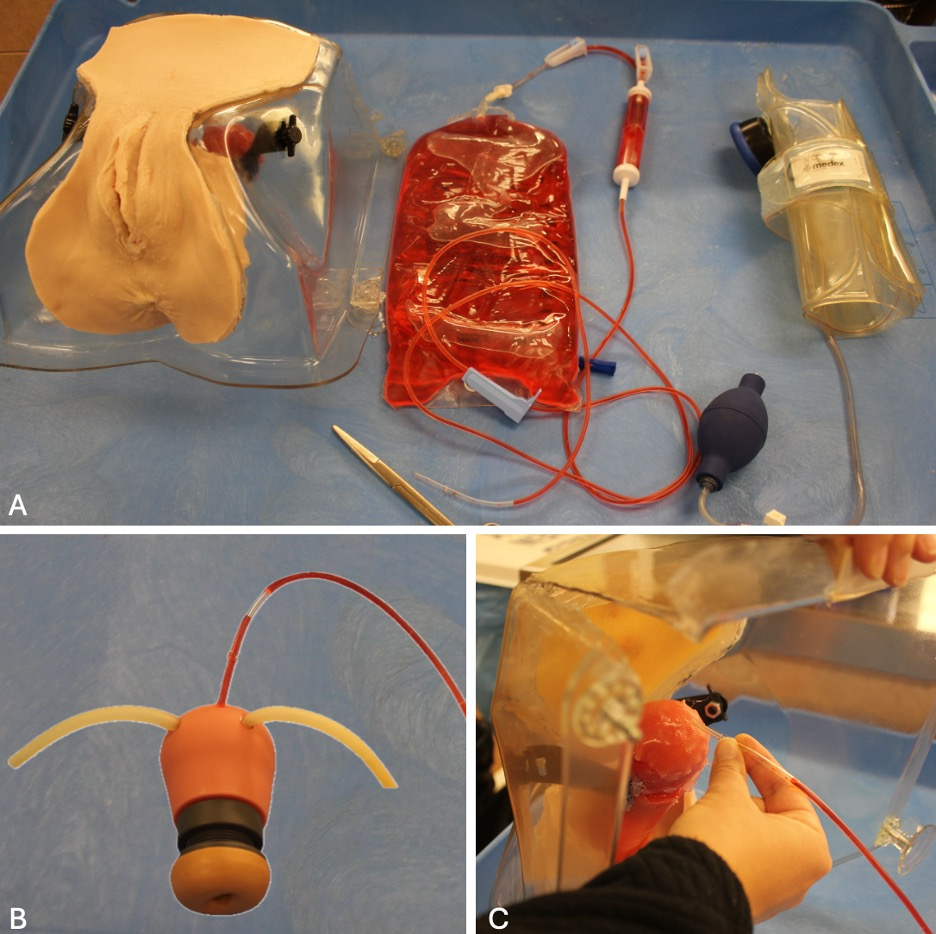


Hemorrhaging uterus model. A) Female pelvis, bag of fake blood, and pressure bag for IV. B) Rubber uterus with patent cervix and puncture for IV tubing to enter uterine fundus. C) IV tubing entering uterine fundus with model attached to female pelvis.
