# Supplementary material for: In-Situ Simulation for Enhancing Safety in Outpatient Hysteroscopy: Development and Evaluation of a Crisis Resource Management-Based Training Package
Source: MedEdPORTAL. 2026 Jun 5;22:11604. doi: 10.15766/mep_2374-8265.11604 (PMC13236966; doi:10.15766/mep_2374-8265.11604)
Supplement: Supplementary file 1 — Oversedation Case.docxHemorrhage Case.docxLAST Case.docxVasovagal Case.docxHemorrhaging Uterus Model.docxDebriefing Materials.docxCrisis Resource Management Primer.docxLatent Safety Threats Template.docxSelf-Efficacy Tool Presurvey.docxSelf-Efficacy Tool Postsurvey.docxParticipant Evaluation Form.docx [file mep_2374-8265.11604-s001.zip › mep_2374-8265.11604-s001/F. Debriefing Materials.docx]

**Appendix F. Debriefing Materials**

The facilitator asks one of the learners to summarize the scenario. They then work through the questions below:

**General Debriefing Questions**

1. Medical Expertise:
   1. What is the clinical progression of this presentation?
   2. What is the relevant differential diagnosis?
   3. What are the risk factors for this presentation?
   4. What is the initial treatment of this presentation?
   5. Scenario specific:
      1. Oversedation- What are the specific antidotes for oversedation based on medications given?
      2. Hemorrhage- What are the specific considerations for acute hemorrhage in the outpatient setting?
      3. LAST- What are the specific considerations for LAST when implementing ACLS protocols?
      4. Vasovagal- What are the specific considerations for a vasovagal episode in the outpatient setting?
2. Team Functioning: With respect to team functioning, what went well? What might be done differently next time? Consider different facets of crisis resource management including:
   1. Leadership
   2. Resource allocation
   3. Situational awareness
   4. Communication
3. Equipment:
   1. Did you have the equipment required easily accessible? If not, what changes would you make to optimize the safety of your outpatient hysteroscopy suite? Consider the following scenario-specific resources:
      1. Oversedation- crash cart, medications (naloxone, flumazenil)
   2. Hemorrhage- intrauterine balloon, crash cart, medications (uterotonics, tranexamic acid)
      1. LAST- crash cart, medications, lipid emulsion
      2. Vasovagal- portable monitors, IV supplies, crash cart, reclining chair or transfer board
   3. Was a cognitive aid used? If so, was it helpful? If not, would it be useful to have one?
4. Human resources: Did you have the human resources necessary to safely respond to the situation? If so, were they any remaining knowledge gaps to be addressed?

**Scenario Specific Considerations**

Oversedation

*Medical Knowledge:*

Intravenous sedation is commonly used for more complex hysteroscopic procedures or in cases of patient anxiety. The most common medications are fentanyl and midazolam. While some centres have anesthetists, this can also be given as physician-led, nurse-administered anesthesia. When patients present with oversedation one must consider the differential diagnosis. The reversal agent for fentanyl is naloxone (0.1-2 mg IV, onset one minute, duration 15-30 minutes), and the reversal agent for benzodiazepines is flumazenil (0.2 mg/min IV, maximum dose 1.0 mg, onset one minute, duration 45 minutes).^14^ It is important to be aware that the duration of the sedative may exceed that of the reversal agent, thus patients require longer observation periods.^14^

*Anticipated Management Mistakes:*

1. Failure to recognize the patient took Ativan: Some learners did not think to ask when medications were last taken on history, thus they did not recognize the cause of oversedation. Even without this knowledge they should be able to provide the necessary supportive care.
2. Unfamiliarity with antidotes: We found most learners were unaware of the antidotes to the IV sedation medications given. We created specific debriefing materials and cognitive aids to cover this information.
3. Inability to recognize the need for prolonged monitoring: In patients with oversedation, even when antidotes are given, the effect of the original drug may outlive the effect of the antidote, causing oversedation to recur. Participants must recognize the patient requires transfer to a higher monitored setting.

Hemorrhage

*Medical Knowledge:*

Hemorrhage is commonly seen in obstetrics, and the same principles can be applied to the gynecology setting. Resuscitative measures must be applied. Providers should consider mechanical compressive maneuvers including bimanual compression, bladder emptying, and placing an intrauterine balloon catheter. Special agents including antifibrinolytic tranexamic acid (1g IV) or uterotonic misoprostol (200-400 µg oral/sublingual) should be easily accessible in the OHS and used as needed.^15^ Uterotonics Ergometrine (250 µg IM) or carboprost (250 µg IM) can also be considered, however these medications must be stored in refrigerated conditions; thus, participants should be aware of where and how to access them if needed.

*Anticipated Management Mistakes:*

1. Unfamiliarity with management of gynecologic hemorrhage: Participants are generally very comfortable managing postpartum hemorrhage, but when the patient is not pregnant there is hesitation and confusion about appropriate medications. We designed a bleeding algorithm as a cognitive aid and include debriefing materials to highlight the similarities and differences between the two clinical situations.
2. Inability to recognize the need for transfer and further management: Some participants felt comfortable once bleeding was stopped but did not consider the next steps in management. This patient requires surgical management in the hospital setting with the resources available to manage high volume blood loss.

LAST

*Medical Knowledge:*

Local anesthetics are used in daily practice, but toxicity can have catastrophic outcomes. If absorbed or injected intravenously patients can experience neurologic and cardiac sequelae.^10^ Neurologic findings include perioral numbness, metallic taste, dizziness, or tinnitus, but can progress to seizures or coma. Cardiac manifestations begin with hypertension and tachycardia but can transition into hypotension, arrhythmias, or cardiac arrest.^10^ Acute treatment includes standard stabilization measures. In addition, practitioners should utilize benzodiazepines for seizure prevention and an IV lipid emulsion, which can act as a “sink” by binding the local anesthetic in the vasculature.^13^ Should the patient decline into cardiac arrest, management varies from standard Advanced Cardiac Life Support (ACLS) protocol, necessitating lower doses of epinephrine and avoiding the use of beta blockers, calcium channel blockers, and vasopressin.^16^ The American Society of Regional Anesthesia and Pain Medicine periodically updates its advisories to manage this complication, and we recommend utilizing their cognitive aids when debriefing this scenario.^16^

*Anticipated Management Mistakes:*

1. Unfamiliarity with ACLS protocols: Not all healthcare providers are required to have ACLS training, and even those that do thankfully use it so rarely that they have less familiarity with the protocols.
2. Unfamiliarity with LAST management: Participants are generally good at identifying early signs of LAST but are unaware of the differences in ACLS protocols when LAST is suspected and the need for lipid emulsion treatment.

Vasovagal

*Medical Knowledge:*

Manipulation of the cervix, pain, and anxiety can all lead to stimulation of the vagal nerve, which can result in a vasovagal episode. The basic approach is the monitor the patient, elevate legs, provide fluids, and ensure no red flags. If bradycardia ensues, refer to the ACLS Bradycardia algorithm.^19^

*Anticipated Management Mistakes:*

1. Difficulty managing patient in the hallway: We found that when moved to an uncomfortable or less familiar environment, the cognitive load increases and learners have difficulty with basic supportive care e.g. assessing vital signs. We modified our debriefing session to include an orientation of available portable resources in the hysteroscopy suite.
