# Supplementary material for: In-Situ Simulation for Enhancing Safety in Outpatient Hysteroscopy: Development and Evaluation of a Crisis Resource Management-Based Training Package
Source: MedEdPORTAL. 2026 Jun 5;22:11604. doi: 10.15766/mep_2374-8265.11604 (PMC13236966; doi:10.15766/mep_2374-8265.11604)
Supplement: Supplementary file 1 — Oversedation Case.docxHemorrhage Case.docxLAST Case.docxVasovagal Case.docxHemorrhaging Uterus Model.docxDebriefing Materials.docxCrisis Resource Management Primer.docxLatent Safety Threats Template.docxSelf-Efficacy Tool Presurvey.docxSelf-Efficacy Tool Postsurvey.docxParticipant Evaluation Form.docx [file mep_2374-8265.11604-s001.zip › mep_2374-8265.11604-s001/G. Crisis Resource Management Primer.docx]

**Appendix G. Crisis Resource Management Primer**

**Crisis Resource Management (CRM)** refers to the non-technical skills that allow healthcare teams to manage emergencies effectively. These skills optimize team performance, decision-making, and patient safety during high-stress situations. CRM focuses on how teams lead, communicate, allocate resources, and maintain awareness during crises.

**1. Leadership**

Effective leadership provides structure, coordination, and direction during a crisis.

Key behaviors:

- Clearly identify a team leader
- Maintain a global overview of the situation
- Delegate tasks rather than performing all actions personally
- Prioritize interventions and guide the overall management plan
- Remain calm and decisive to support team function

Good leaders balance hands-off coordination with clinical oversight, ensuring the team works efficiently toward shared goals.

**2. Resource Allocation**

Effective use of all available resources: personnel, equipment, and cognitive bandwidth.

Key behaviors:

- Delegate tasks appropriately to prevent overload
- Assign specific roles (e.g., airway, medications, documentation)
- Use the skills and experience of all team members
- Call for additional help early when needed
- Ensure equipment and medications are readily available

Optimal resource allocation prevents errors that arise from task saturation or under-utilized team members.

**3. Situational Awareness**

Situational awareness refers to maintaining a continuous understanding of the patient, environment, and evolving clinical situation.

Key behaviors:

- Monitor the patient’s condition and response to treatment
- Maintain awareness of team actions and workload
- Anticipate potential complications or next steps
- Avoid fixation errors—do not focus on one problem while missing others
- Periodically reassess: *“What is happening now? What might happen next?”*

Maintaining situational awareness allows teams to detect problems early and adapt management plans quickly.

**4. Communication**

Clear communication is essential for coordinating team actions and preventing misunderstandings.

Key behaviors:

- Use direct, explicit communication
- Provide succinct, effective handover (eg. SBAR – situation, background, assessment, recommendation)
- Assign tasks using names and specific instructions
- Practice closed-loop communication (instruction → confirmation → completion)
- Encourage speaking up if concerns arise
- Share updates so the team maintains a shared mental model

Together, these CRM principles improve team coordination, error detection, and patient safety during medical emergencies.
