# Supplementary material for: In-Situ Simulation for Enhancing Safety in Outpatient Hysteroscopy: Development and Evaluation of a Crisis Resource Management-Based Training Package
Source: MedEdPORTAL. 2026 Jun 5;22:11604. doi: 10.15766/mep_2374-8265.11604 (PMC13236966; doi:10.15766/mep_2374-8265.11604)
Supplement: Supplementary file 1 — Oversedation Case.docxHemorrhage Case.docxLAST Case.docxVasovagal Case.docxHemorrhaging Uterus Model.docxDebriefing Materials.docxCrisis Resource Management Primer.docxLatent Safety Threats Template.docxSelf-Efficacy Tool Presurvey.docxSelf-Efficacy Tool Postsurvey.docxParticipant Evaluation Form.docx [file mep_2374-8265.11604-s001.zip › mep_2374-8265.11604-s001/H. Latent Safety Threats Template.docx]

**Appendix H. Latent Safety Threats Template**

| **Scenario** | **Knowledge** | **Physical Resources** | **CRM** | **Intervention** |
| --- | --- | --- | --- | --- |
| General |  |  |  |  |
| 1. Oversedation |  |  |  |  |
| 1. Hemorrhage |  |  |  |  |
| 1. LAST |  |  |  |  |
| 1. Vasovagal |  |  |  |  |

*Example:*

| ***Scenario*** | ***Knowledge*** | ***Physical Resources*** | ***CRM*** | ***Intervention*** |
| --- | --- | --- | --- | --- |
| *LAST* | - *Team unaware of LAST management protocols* | - *Nurse too short to do adequate chest compressions* - *Lipid emulsions not available on unit* | - *Handover- SBAR* - *Sharing mental model/verbalization- “I thought that but never said it”* - *Cognitive aid use* | - *Post LAST algorithm on wall* - *Stool in room for CPR* - *Lipid emulsion- check with pharmacy re: stocking on unit or location in hospital and protocol to access* |
