# Supplementary material for: In-Situ Simulation for Enhancing Safety in Outpatient Hysteroscopy: Development and Evaluation of a Crisis Resource Management-Based Training Package
Source: MedEdPORTAL. 2026 Jun 5;22:11604. doi: 10.15766/mep_2374-8265.11604 (PMC13236966; doi:10.15766/mep_2374-8265.11604)
Supplement: Supplementary file 1 — Oversedation Case.docxHemorrhage Case.docxLAST Case.docxVasovagal Case.docxHemorrhaging Uterus Model.docxDebriefing Materials.docxCrisis Resource Management Primer.docxLatent Safety Threats Template.docxSelf-Efficacy Tool Presurvey.docxSelf-Efficacy Tool Postsurvey.docxParticipant Evaluation Form.docx [file mep_2374-8265.11604-s001.zip › mep_2374-8265.11604-s001/J. Self-Efficacy Tool Postsurvey.docx]

**Appendix J. Self-Efficacy Evaluation Tool for Outpatient Gynecologic Emergencies: POST-Survey**

1. I feel comfortable in managing emergencies in the outpatient setting:

*False Neutral True*

1 2 3 4 5

1. I know what resources are available in emergency situations in the outpatient setting:

*False Neutral True*

1 2 3 4 5

1. I can appropriately use the available resources in emergencies in the outpatient setting:

*False Neutral True*

1 2 3 4 5

1. I communicate well in emergency situations:

*False Neutral True*

1 2 3 4 5

1. I am competent in managing the following emergencies in an outpatient setting:

|  | 1  No knowledge, unable to perform | 2  Some knowledge, but need a lot of guidance | 3  Basic knowledge, but guidance still needed | 4  Reasonably confident, some guidance needed | 5  Highly knowledgeable and confident, independent |
| --- | --- | --- | --- | --- | --- |
| Oversedation |  |  |  |  |  |
| Acute Hemorrhage |  |  |  |  |  |
| Local Anesthetic Systemic Toxicity |  |  |  |  |  |
| Vasovagal Episode |  |  |  |  |  |

1. I am competent in employing the following technical skills in an outpatient setting:

|  | 1  No knowledge, unable to perform | 2  Some knowledge, but need a lot of guidance | 3  Basic knowledge, but guidance still needed | 4  Reasonably confident, some guidance needed | 5  Highly knowledgeable and confident, independent |
| --- | --- | --- | --- | --- | --- |
| Bag Mask Ventilation |  |  |  |  |  |
| Performing CPR |  |  |  |  |  |
| Inserting Intrauterine Balloon for Tamponade |  |  |  |  |  |
| Administering emergency IV medications |  |  |  |  |  |

Adapted from: Self-efficacy rating scale used by residents and fellows to determine self-reported improvement in emergency scenario management pre- and postcurriculum. OB, Obstetrics. Espey et al. Outpatient emergency simulation curriculum. Am J Obstet Gynecol 2017.^10^
