# Supplementary material for: In-Situ Simulation for Enhancing Safety in Outpatient Hysteroscopy: Development and Evaluation of a Crisis Resource Management-Based Training Package
Source: MedEdPORTAL. 2026 Jun 5;22:11604. doi: 10.15766/mep_2374-8265.11604 (PMC13236966; doi:10.15766/mep_2374-8265.11604)
Supplement: Supplementary file 1 — Oversedation Case.docxHemorrhage Case.docxLAST Case.docxVasovagal Case.docxHemorrhaging Uterus Model.docxDebriefing Materials.docxCrisis Resource Management Primer.docxLatent Safety Threats Template.docxSelf-Efficacy Tool Presurvey.docxSelf-Efficacy Tool Postsurvey.docxParticipant Evaluation Form.docx [file mep_2374-8265.11604-s001.zip › mep_2374-8265.11604-s001/K. Participant Evaluation Form.docx]

**Appendix K. Simulation Patient Safety Program – Participant Evaluation Form**

Profession:

Registered Nurse O RPN O NP O Nursing Student O

Respiratory Therapist O Med Student O Resident O Fellow O Physician O

PCA O Administrative Assistant O X-ray Tech O Other O

Objectives:

Participants in the SPSP in-situ simulation program will:

- Embrace the principles of crisis resource management, specifically: closed loop communication, summarizing, task assignment, leadership and followership;
- Manage critical incidents in their clinical setting;
- Identify latent threats to quality and safety in their clinical environment.

Evaluation:

1= strongly disagree, 5= strongly agree

Were the stated learning objectives achieved?

1 2 3 4 5

Was the simulation relevant to your practice?

1 2 3 4 5

Did you learn about COLLABORATION within a team?

1 2 3 4 5

Did you learn about COMMUNICATION within a team?

1 2 3 4 5

Was the activity free from bias?

1 2 3 4 5

Was the amount of time allotted to this session sufficient?

1 2 3 4 5

Comments:

How can we make this program even more valuable?

How will your experience today change your practice?
