# Supplementary material for: In-Situ Simulation for Enhancing Safety in Outpatient Hysteroscopy: Development and Evaluation of a Crisis Resource Management-Based Training Package
Source: MedEdPORTAL. 2026 Jun 5;22:11604. doi: 10.15766/mep_2374-8265.11604 (PMC13236966; doi:10.15766/mep_2374-8265.11604)
Supplement: Supplementary file 1 — Oversedation Case.docxHemorrhage Case.docxLAST Case.docxVasovagal Case.docxHemorrhaging Uterus Model.docxDebriefing Materials.docxCrisis Resource Management Primer.docxLatent Safety Threats Template.docxSelf-Efficacy Tool Presurvey.docxSelf-Efficacy Tool Postsurvey.docxParticipant Evaluation Form.docx [file mep_2374-8265.11604-s001.zip › mep_2374-8265.11604-s001/_Educational Summary Report_11604.pdf]

MedEdPORTAL
THE AAMC JOURNAL OF TEACHING AND LEARNING RESOURCES

Original Publication
Open Access

In-Situ Simulation for Enhancing Safety in Outpatient Hysteroscopy: Development and Evaluation of a Crisis Resource Management-Based Training Package

Chelsie Warshafsky, MD, MS*, Sukhbir Sony Singh, MD, Adam B. Garber, MD, MSc

*Corresponding author: chelsie.warshafsky@utoronto.ca

Abstract

Introduction: Hysteroscopy is a common gynecologic procedure increasingly performed in outpatient settings, yet in Canada cases still occur in operating rooms. As care transitions to ambulatory suites, processes for managing complications remain poorly defined. This educational initiative aimed to design in-situ simulation scenarios to enhance patient safety during outpatient hysteroscopy by strengthening team preparedness and identifying system gaps. **Methods:** A needs assessment was conducted via literature review and interviews with local staff. Findings informed the creation of 4 simulation scenarios targeting the most common and catastrophic adverse events in the outpatient setting: intravenous sedation overdose, hemorrhage, local anesthetic toxicity, and vasovagal reaction. Physicians and nurses working in the outpatient suite were invited to participate. Surveys assessing self-efficacy components on a 5-point Likert scale were administered pre- and postsimulation. **Results:** Existing literature revealed minimal simulation-based training for outpatient hysteroscopy. Twelve participants (8 physicians, 4 nurses), with experience ranging from 6 months to 23 years, engaged in 4 simulation sessions. Across all measured domains, participants reported improved confidence in managing complications. **Discussion:** Simulation effectively prepares teams for rare, high-acuity events but remains underutilized in ambulatory gynecology. These scenarios can be deployed in simulation centers to enhance crisis resource management or in-situ to uncover latent safety threats and test system readiness. They offer a structured approach to support the safe establishment of outpatient hysteroscopy services and can serve as a continuing quality assurance tool. Implementing standardized, team-based simulation may help close safety gaps as outpatient hysteroscopy becomes the preferred standard of care.

Keywords
Simulation, Hysteroscopy, Crisis Resource Management, In-Situ Simulation, Outpatient Procedures, OB/GYN, Critical Care Medicine

Educational Objectives

By the end of this activity, learners will be able to:

1. Recognize the presenting signs and symptoms of the most common and catastrophic hysteroscopy complications.
2. Initiate management of complications in an outpatient surgical suite.
3. Appraise existing equipment and unit protocols.
4. Identify latent safety threats in response to complications in the outpatient hysteroscopy setting.
5. Apply the principles of crisis resource management when managing outpatient hysteroscopic emergencies.

Citation:
Warshafsky C, Sing SS, Garber, AB. In-situ simulation for enhancing safety in outpatient hysteroscopy: development and evaluation of a crisis resource management-based training package. *MedEdPORTAL.* 2026;22:11604.
https://doi.org/10.15766/mep_2374-8265.11604

Introduction

Hysteroscopy, or the process of visualizing the uterine cavity with access through the cervix, allows surgeons to "see and treat" a multitude of intracavitary pathologies. It is the gold standard diagnostic and therapeutic measure for conditions including abnormal uterine bleeding, structural causes of infertility, retained products of conception, uterine anomalies, and more.[1] Many national and international guidelines indicate that the ideal location for such procedures is in the outpatient or ambulatory setting.[2,3] The use of local anesthetic and/or intravenous sedation, instead of general anesthesia, in addition to adjuncts focusing on patient comfort and anxiety, allows for increased access, faster recovery, and greater patient satisfaction in

Copyright © 2026 Warshafsky et al. This is an open-access publication distributed under the terms of the Creative Commons Attribution license.

comparison to the traditional operating room approach.[3] Despite increasing evidence of its feasibility and economic benefits, most hysteroscopies in Canada are still performed in the operating room.[2,3] A recent position paper by the Canadian Society for the Advancement of Gynecologic Excellence (CanSAGE) outlined the need for the migration of hysteroscopy to the ambulatory setting.[4] With more centers adopting an office approach, ensuring a safe setting is paramount.

Crisis resource management (CRM), originally developed in aviation, was introduced into medical training through anesthesiology in 1980.[5] CRM aims to strengthen team functioning by addressing a set of principles focusing on cognitive and interpersonal skills. With multiple reports indicating that most maternal mortality is due to preventable causes, CRM principles have long been applied in the obstetrics setting via simulation-based education and yielded positive results.[6] Studies have shown that simulation training increases provider confidence, resource utilization, communication, and management in emergent situations.[7] It has also been shown to improve team competence as a whole.[7]

Outpatient procedure suites offer significant benefits but in certain cases may be operating with less support, resources, personnel, and equipment compared to the hospital setting, thereby increasing the stakes if complications occur.[6] Simulation-based education is ideal to promote the principles of CRM. In fact, in 2010, the American College of Obstetricians and Gynecologists (ACOG) Task Force on Patient Safety in the Office Setting recommended quarterly simulation drills to maintain knowledge and skills for the infrequent but high-risk emergencies.[8] Despite this, there are few resources in the literature to implement this practice. There is a multitude of data on simulation in hysteroscopy, but it largely focuses on technical skills acquisition.[9] Two papers identified common outpatient hysteroscopic emergencies, outlining clinical vignettes and providing frameworks for approach based on CRM skills.[7,10] One additional paper actually developed a simulation curriculum and implemented it with residents, focusing on self-efficacy and critical actions performance in a simulation lab.[6] However, there are no resources available to our knowledge that describe easily implementable in-situ simulation scenarios, allowing teams to assess medical knowledge, CRM, and latent safety threats.

The objective of this educational innovation was to create, evaluate, and disseminate a package of in-situ simulation scenarios to optimize patient safety in an outpatient hysteroscopy setting by promoting team competence and systems assessment. The target audience for this innovation involves interprofessional teams working in the outpatient procedural setting, including nurses, residents, and practicing gynecologists.

Methods

Development

We performed a comprehensive literature review, investigating the existing curriculum for outpatient procedures in general, hysteroscopy in particular, and associated medical crises. We also interviewed local interprofessional team members at The Ottawa Hospital working in our own high-volume outpatient hysteroscopy suite (OHS) with the following questions:

1. What types of emergencies have you seen in the OHS?
2. What are the rare but dangerous cases you feel unequipped to manage in the OHS?
3. If we were to run in-situ simulations in the OHS, what types of scenarios do you think would be beneficial?

Literature review indicated a paucity of existing simulation curriculum for outpatient hysteroscopy. Studies have looked at technical skills simulation alone, and 2 studies addressed emergencies in the outpatient setting.[11,12] However, no programs focused on both team training and auditing existing safety systems. Based on this, and responses to the preceding questions, we designed cases that could be done in-situ in the OHS setting with objectives for both team training and system improvement. We chose the 4 scenario topics to address 2 of the most common and 2 of the most catastrophic complications encountered in the outpatient hysteroscopy setting, including oversedation, hemorrhage, local anesthetic systemic toxicity (LAST), and vasovagal episode.

Using rigorous scenario design principles and the structure of previously published outpatient simulation scenarios,[12,13] we created the preceding 4 scenarios (Appendices A-D). For each scenario, we defined learning objectives and, based on these, created patient cases. We outlined equipment requirements and made a simple model for the Hemorrhage scenario (Appendix E). We defined detailed timelines to allow for a clear case progression (see Appendices A-D). We designed each scenario to resolve within 15 minutes, with up to 45 minutes for debriefing, allowing for completion of scenario-specific learning objectives within 1 hour. The entire training package took 4 hours to complete but could be run in various hourly increments depending on time available.

Copyright © 2026 Warshafsky et al. This is an open-access publication distributed under the terms of the Creative Commons Attribution license.

We designed the scenarios to target learners from the start of specialty training to seasoned practitioners. Although our initial trial only included staff actively working in our OHS, each scenario was linked to a Royal College of Physicians and Surgeons of Canada Entrustable Professional Activity for Obstetrics & Gynecology residents for easy transition to use in the trainee setting.[13] This project was submitted to the Ottawa Health Science Network Research Ethics Board. It was determined that the project fell within context of quality initiative, quality improvement, quality assurance, and/or program evaluation. Consequently, a full review was not required.

Equipment/Environment

The following equipment is recommended for successful implementation of the simulation series, in general:

- Patient simulator (ACLS manikin)
- Monitors
  - Noninvasive blood pressure cuff
  - Pulse oximeter
- Routine hysteroscopy tray and setup
  - 20cc syringe marked with specific local anesthetic used at your center (including concentration +/– epinephrine)
- Venous access supplies
  - Peripheral IV catheters
  - 1L bag of crystalloid solution
- Oxygen supplies
  - Nasal cannula
  - Simple face mask
  - Ambu bag and mask
- Crash cart (left in usual location)
  - ACLS medications
  - Defibrillator
  - Airway supplies (laryngoscope, endotracheal tube)

Oversedation scenario equipment: Medication syringes labeled as (1) Fentanyl, (2) Midazolam, (3) Naloxone, (4) Flumazenil

Hemorrhage scenario equipment: Uterus model allowing for insertion of hysteroscope and Foley balloon (see Appendix E); Bag of fake blood and IV tubing with cannula to bleed from uterus; Uterotonics; Tranexamic acid; Foley balloon

LAST scenario equipment: Lipid emulsion - diluted milk

Vasovagal scenario equipment: None specific

Personnel

Each scenario requires a minimum of 1 gynecologist and 1 nurse. However, we had 2 nurses, as our OHS regularly has a circulating and scrub nurse for procedures. As well, due to scheduling, we often had 2 staff gynecologists present, thus one acted as the primary surgeon and the other as assist, though this role could easily be delegated to a trainee if present. Participants traded roles for the second scenario to provide a more well-rounded experience. Facilitators were simulation-trained practicing gynecologists. One facilitator provided relevant information requested and prompted participants as needed, whereas the other acted as the voice of the patient. A simulation technician was responsible for executing vital sign changes.

Implementation

The scenarios were performed in our own OHS as part of the larger Simulation Patient Safety Program (SPSP) implemented across departments at the University of Ottawa (used with permission). The objectives of the SPSP are the following:

1. Embrace the principles of CRM, specifically closed-loop communication, summarizing, task assignment, leadership, and followership.
2. Manage critical incidents in their clinical setting.
3. Identify latent threats to quality and safety in their clinical environment.

The facilitators briefed participants on the setup and objectives of the day, then introduced the first scenario. Participants were subsequently moved to the procedure room where the manikin was set up with the equipment mentioned previously. The facilitator providing the patient voice was positioned near the patient's head, hidden from view, to aid in realism. The other facilitator and simulation technician were stationed in the corner of the procedure room, out of the way from activity but able to observe the participants and communicate as needed. Each scenario took approximately 15 minutes to complete. After the first scenario was completed, the participants moved to a different room where they partook in a debrief with the 2 facilitators for approximately 45 minutes while the simulation technician set up for the next scenario in the procedure room. The facilitators then briefed the participants on the second scenario, and the preceding was repeated. The day concluded with all participants identifying 1 major takeaway point from the day that they would bring forward into their practice.

The scenarios were grouped with Oversedation and Hemorrhage (see Appendices A and B) done on one day, and LAST and Vasovagal (see Appendices C and D) done another day, due to ease of scheduling. The scenarios were grouped this way such that 1 common and 1 rare scenario were run per day.

Copyright © 2026 Warshafsky et al. This is an open-access publication distributed under the terms of the Creative Commons Attribution license.

Debriefing

Debriefing questions were created to ensure that specific discussion points were addressed and objectives were met (Appendix F). While debrief sessions are often open-ended, with trained debriefers guiding the discussion based on how the scenario played out, we chose to define these questions so that any health care provider could employ this user-friendly guide and ensure that its full potential is realized. The debriefing questions focused on medical expertise, team functioning, equipment, and human resources. Although more exhaustive resources exist on CRM principles, a 1-page CRM Primer[14] (Appendix G) was included to aid in the debrief. In addition, the Latent Safety Threats Template (Appendix H) was used to help identify threats in each scenario that could be addressed. This was completed as topics came up during the debriefing conversation.

Assessment

Prior to each session, the participants completed the Self-Efficacy Evaluation Tool Presurvey (Appendix I), which assessed their knowledge and comfort in managing outpatient hysteroscopy emergencies in the areas of CRM, scenario-specific knowledge, and technical skills. Following each session (in which 2 scenarios were completed, as described earlier), participants completed the Self-Efficacy Evaluation Tool Postsurvey (Appendix J). The self-efficacy surveys were adapted from a previously validated survey and modified to assess our specific scenarios.[6] Pre- and postintervention self-efficacy scores were analyzed using paired *t* tests for each domain, with results reported as means. Statistical significance was defined as $P < .05$. Additionally, the SPSP participant evaluation form was completed (Appendix K), which gauged the utility of the simulation and provided an opportunity to respond to open-ended questions.

Critical actions were assessed during the scenarios and are indicated in the Learner Actions column of each case progression table (see Appendices A-D). These actions were identified through reviewing the management of each presentation and identifying the critical components required to safely move the case forward.[15-18] The first segment of the debriefing questions focused on medical expertise, at which time critical actions were discussed and formative feedback was provided.

Results

The scenarios were initially piloted in a single day with 4 nurses and 2 physicians. During this session, it became clear that doing all 4 scenarios in 1 day was too much from both a time commitment and participant fatigue perspective. Scenarios were adjusted according to feedback from the pilot session. Four simulation sessions were then completed, with 2 scenarios per 2-hour session, as described previously. All OHS staff participated in this educational initiative, totaling 8 physicians and 4 nurses. Due to the ratio of staffing, each physician completed 2 scenarios and nurses completed all 4 scenarios. Level of experience ranged from 6 months to 23 years.

The self-assessment surveys were completed at the start and end of each 2-hour session. Survey response rate was 100%. Participants demonstrated significant improvements in multiple domains following simulation training. Mean self-efficacy scores increased for comfort in managing emergencies (3.23 vs 4.08, $P = .002$), resource knowledge (3.38 vs 4.46, $P < .001$), resource utilization (3.58 vs 4.15, $P = .009$), communication (3.92 vs 4.31, $P = .033$), oversedation management (3.00 vs 3.56, $P = .021$), and management of LAST (3.00 vs 4.25, $P < .001$). Improvements in hemorrhage management demonstrated a positive trend but did not reach statistical significance. No significant changes were observed in cardiopulmonary resuscitation or uterine balloon tamponade, likely reflecting high baseline confidence and a ceiling effect. The Figure demonstrates the change in pre- and postsimulation self-efficacy.

The Table indicates the aggregated responses to the SPSP Participant Evaluation Form (see Appendix K) completed for each session. Additionally, participants were asked 2 open-ended questions. First, they were asked, “How can we make this program even more valuable?” Participants unanimously indicated that participating in these simulation sessions was incredibly valuable, and there were multiple requests to run the sessions more frequently. Second, participants were asked, “How will your experience today change your practice?” Responses generally addressed CRM skills, including “more confidence,” “improved team communication/collaboration,” “more knowledgeable,” “feel more comfortable in emergent situations,” and “more awareness of our own resources.”

Latent safety threats were identified in all categories, and changes to both the OHS and unit protocols were made based on the identified safety threats.

The cases have also been implemented with practicing gynecologists attending a conference on hysteroscopy (Canadian Hysteroscopy Practice and Simulation [CHyPS] Course) in both 2022 and 2024 in the simulation center, in groups up to 8 participants. The purpose of this session was to demonstrate to how to run the simulations and implement them in one’s own practice setting. Some participants acted in the simulation

Copyright © 2026 Warshafsky et al. This is an open-access publication distributed under the terms of the Creative Commons Attribution license.

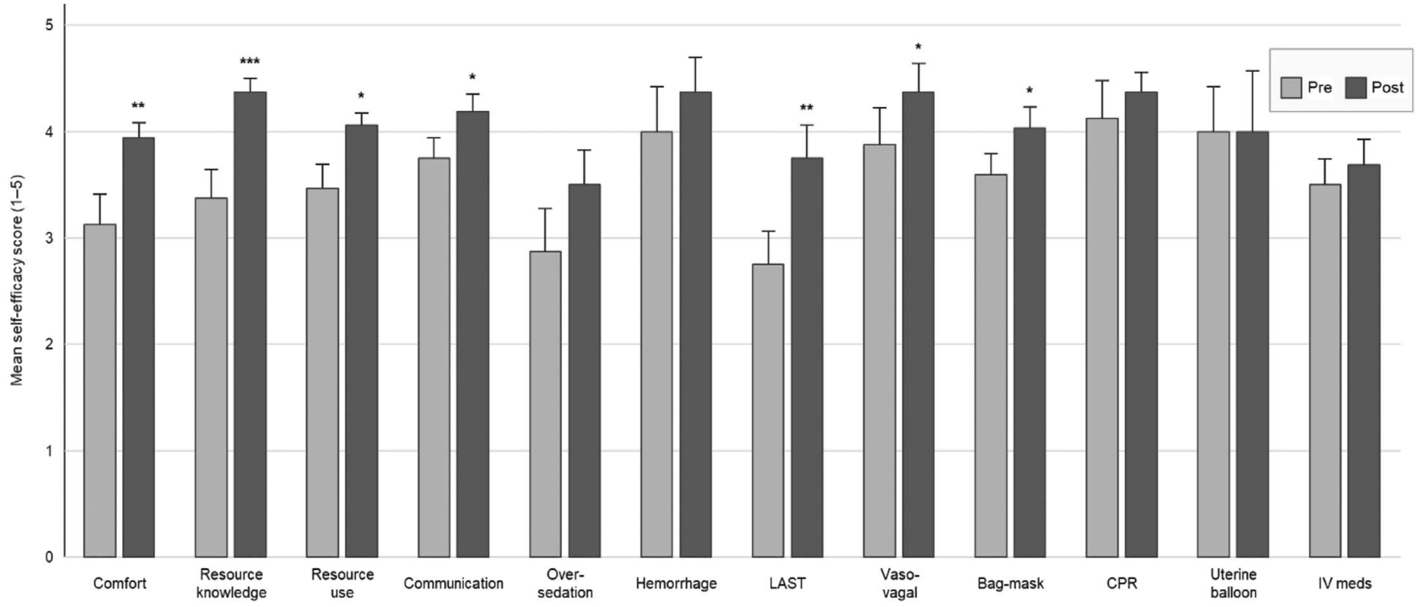

Figure. Pre- and postsimulation self-efficacy scores. Mean self-efficacy scores are shown before and after simulation training across crisis management and technical skill domains. Error bars indicate standard error. Asterisks denote statistically significant differences by paired *t* test (*P* < .05, **P* < .01, ***P* < .001, ****P* < .0001). Abbreviations: CPR, cardiopulmonary resuscitation; IV meds, intravenous medications; LAST, local anesthetic systemic toxicity.

while others were provided with the debriefing materials (see Appendix F) while watching the scenario unfold and taught how to use the tools during the debrief sessions. Given the different objective and setting in the case of the CHyPS course, the surveys described previously were not administered, as they were not applicable. However, feedback from participants on this session was overwhelmingly positive, with comments focusing on the importance of running these simulations at their local centers.

Discussion

These simulations addressed critical aspects of outpatient hysteroscopy, including medical knowledge, CRM skills, and system safety threats. They were designed to be practical, engaging, and adaptable to real-world conditions, with a focus on interprofessional collaboration. Each scenario met learning objectives, and feedback confirmed the value of this training (see Table). Self-efficacy assessments indicated a significant improvement across all nontechnical CRM skills (see Figure).

Table. Simulation Patient Safety Program Participant Evaluation Responses

Evaluation	Score[a]
Were the stated learning objectives achieved?	4.94
Was the simulation relevant to your practice?	4.97
Did you learn about collaboration within a team?	4.97
Did you learn about communication within a team?	5.00
Was the activity free from bias?	5.00
Was the amount of time allotted to this session sufficient?	5.00

[a]Rated on a 5-point scale (1 = *strongly disagree*, 5 = *strongly agree*).

The selected scenarios represent both common and critical adverse events in outpatient hysteroscopy. Oversedation (see Appendix A) allowed participants to review medication antidotes, a vital understanding in settings without anesthesia support. Hemorrhage (see Appendix B) emphasized the specific needs of gynecologic bleeding management and how it differs from obstetrical hemorrhage. LAST (see Appendix C) ensured that participants practice cardiac life support protocols and test the crash cart equipment. While in the hospital cardiac emergencies are generally managed by our critical care colleagues, in the outpatient setting the onus may be on the gynecologist as the most responsible physician. Finally, the Vasovagal scenario (see Appendix D) forced participants to troubleshoot this seemingly simple but practically challenging situation, and review transfer and recovery protocols. Each scenario also reinforced the importance of understanding emergency transport procedures, promoting preparedness for transferring patients in the outpatient setting.

Limitations of this resource include the need for physical material resources to conduct the simulations; however, they can be accomplished as described even in a lower resource setting. For instance, vital signs can simply be reported to patients or written on a piece of paper, and Appendix E describes low-fidelity options to simulate a hemorrhaging uterus. More data is arising showing the use of Visually Enhanced Mental Simulation, and this can be used instead of a simulation manikin.[19] Completing

Copyright © 2026 Warshafsky et al. This is an open-access publication distributed under the terms of the Creative Commons Attribution license.

the scenarios in-situ allows for the assessment of resources and latent safety threats but must be balanced against the ongoing demand of clinical activities in the space. Another limitation is the small number of roles, seemingly restricting the number of participants. However, as implemented in the CHyPS course, participants can be divided into actors and debriefers. This allows for more participants and can be employed in larger groups such as residency cohorts. Additionally, the participating physicians and nurses were drawn from the same OHS. While this reflects an established clinical team, members had varying experience working together, and communication and interprofessional coordination challenges still emerged during simulation exercises. This highlights the ongoing value of team-based simulation even within familiar teams.

A final limitation of this educational initiative is the small amount of data currently available to demonstrate a measurable benefit. The findings are also subject to potential bias inherent in self-reported surveys, including repetition effects and survey fatigue, which may influence participant responses. We plan to undertake a more rigorous evaluation of the program's effects and outcomes. Future directions include conducting follow-up surveys to assess knowledge retention and the extent to which CRM principles are applied in the clinical workspace. Additional simulation scenarios are also planned at regular intervals to reinforce learning and maintain preparedness. Expanding participation to include residents and pursuing multi-institutional studies may further strengthen the evidence base and improve the generalizability of this educational approach.

This curriculum has significant implications for both medical education and quality improvement. Running the scenarios in a theater-based simulation setting allows participants to focus on medical knowledge and CRM skills, and could be used for learners of all levels, from residents to experienced staff clinicians. Moving to the in-situ environment adds a level of realism and a quality improvement focus. While still covering medical knowledge and CRM skills, the debrief shifts to include the team's reflections on physical resources and environmental barriers. This facilitates identification of actionable improvements to the clinical environment, leading to enhanced patient safety. Ongoing drills and assessment can then be incorporated into a regular quality assurance initiative.

Given the growing trend of shifting gynecologic procedures to outpatient settings, structured simulations like these play a crucial role in ensuring safety. Regular simulations should be a requirement for setting up outpatient procedure centers and maintaining ongoing patient safety. This comprehensive 4-scenario curriculum offers a 1-stop document that can be utilized to train staff, monitor the safety of existing outpatient hysteroscopy programs, and aid in the design of new outpatient hysteroscopy programs.

Appendices

A. Oversedation Case.docx
B. Hemorrhage Case.docx
C. LAST Case.docx
D. Vasovagal Case.docx
E. Hemorrhaging Uterus Model.docx
F. Debriefing Materials.docx
G. Crisis Resource Management Primer.docx
H. Latent Safety Threats Template.docx
I. Self-Efficacy Tool Presurvey.docx
J. Self-Efficacy Tool Postsurvey.docx
K. Participant Evaluation Form.docx

All appendices are peer reviewed as integral parts of the Original Publication.

Chelsie Warshafsky, MD, MS: Assistant Professor, Faculty of Medicine, University of Toronto; ORCID: https://orcid.org/0000-0002-8268-9141

Sukhbir Sony Singh, MD: Professor, Faculty of Medicine, University of Ottawa

Adam B. Garber, MD, MSc: Associate Professor, Faculty of Medicine, University of Ottawa

Acknowledgments
We would like to acknowledge the team at the University of Ottawa Skills and Simulation Centre for their hard work and dedication to this project.

Disclosures
None to report.

Funding/Support
Dr. Singh has received grants from Pfizer and EndoDiag for clinical research. He has received honoraria for Continuing Professional Development events from Pfizer, Abbvie, and Ethicon.

Prior Presentations
Warshafsky C, Singh SS, Garber AB. Prepare for the worst to deliver the best: promoting safety in an outpatient hysteroscopy setting through simulation based team training. Paper presented at: Harry Oxorn Postgraduate Medical Education Research Day; May 2023; Ottawa, ON.

Copyright © 2026 Warshafsky et al. This is an open-access publication distributed under the terms of the Creative Commons Attribution license.

Warshafsky C, Singh SS, Garber AB. Prepare for the worst to deliver the best: promoting safety in an outpatient hysteroscopy setting through simulation based team training. Paper presented at: Global Congress of Hysteroscopy; April 2023; Sitges, Barcelona, Spain.

Warshafsky C, Singh SS, Garber AB. Prepare for the worst to deliver the best: promoting safety in an outpatient hysteroscopy setting through simulation based team training. Paper presented at: Meridith Marks Day; March 2023; Ottawa, ON.

Warshafsky C, Singh SS, Garber AB. Prepare for the worst to deliver the best: promoting safety in an outpatient hysteroscopy setting through simulation based team training. Paper presented at: CanSAGE 7th Annual Conference; September 2022; Halifax, NS.

Ethical Approval
Ottawa Health Science Network Institutional Review Board reviewed this project.

References

1. Salazar CA, Isaacson KB. Office operative hysteroscopy: an update. *J Minim Invasive Gynecol*. 2018;25(2):199-208. https://doi.org/10.1016/j.jmig.2017.08.009
2. Bennett A, Lepage C, Thavorn K, et al. Effectiveness of outpatient versus operating room hysteroscopy for the diagnosis and treatment of uterine conditions: a systematic review and meta-analysis. *J Obstet Gynaecol Can*. 2019;41(7):930-941. https://doi.org/10.1016/j.jogc.2018.10.002
3. Lee CE, Epp A. Safety and efficiency in a Canadian outpatient gynaecological surgical centre. *J Obstet Gynaecol Can*. 2018; 40(4):426-431. https://doi.org/10.1016/j.jogc.2017.07.027
4. Thiel J, Warshafsky C, Ngan TY, et al. The migration of hysteroscopy from the operating room to an ambulatory setting. *Can J Surg*. 2026;69(1):E68-E70. https://doi.org/10.1503/cjs.008825
5. Bogne Kamdem V, Daelemans C, Englert Y, Morin F, Sansregret A. Using simulation team training with human's factors components in obstetrics to improve patient outcome: a review of the literature. *Eur J Obstet Gynecol Reprod Biol*. 2021;260: 159-165. https://doi.org/10.1016/j.ejogrb.2021.03.015
6. Espey E, Baty G, Rask J, Chungtuyco M, Pereda B, Leeman L. Emergency in the clinic: a simulation curriculum to improve outpatient safety. *Am J Obstet Gynecol*. 2017;217(6): 699.e1-699.e13. https://doi.org/10.1016/j.ajog.2017.09.008
7. Roman RA, Roberts CC, Booth R, Nezhat C, Bhagavath B, Lindheim SR. Crisis management of hysteroscopic surgical complications in the office setting. *Obstet Gynecol Surv*. 2021;76(6):345-352. https://doi.org/10.1097/OGX.0000000000000900
8. Erickson TB, Kirkpatrick DH, DeFrancesco MS, Lawrence HC. Executive summary of the American College of Obstetricians and Gynecologists Presidential Task Force on Patient Safety in the Office Setting: reinvigorating safety in office-based gynecologic surgery. *Obstet Gynecol*. 2010;115(1):147-151. https://doi.org/10.1097/AOG.0b013e3181c4f966
9. Abrahamsen KH, Nielsen MS, Brandt NB, et al. Training and assessment of competencies in hysteroscopy – a systematic review. *Eur J Obstet Gynecol Reprod Biol*. 2026;318:114890. https://doi.org/10.1016/j.ejogrb.2025.114890
10. Schwartz K, Flyckt R, Kim ST, Lindheim SR. Teaming in the ambulatory surgical space and crisis management strategies. *Fertil Steril*. 2022;117(1):22-26. https://doi.org/10.1016/j.fertnstert.2021.09.035
11. Torsher LC, Craigo P, Lynch JJ, Smith HM. Regional anesthesia emergencies. *Simul Healthc*. 2009;4(2):109-113. https://doi.org/10.1097/SIH.0b013e3181925219
12. Cropsey CL, McEvoy MD. Local anesthetic systemic toxicity in a nonoperative location. *Simul Healthc*. 2015;10(5):326-328. https://doi.org/10.1097/SIH.0000000000000110
13. *Entrustable Professional Activities for Obstetrics and Gynecology*. Royal College of Physicians and Surgeons of Canada. Updated September 2024. Accessed March 25, 2026. https://www.royalcollege.ca/content/dam/documents/ibd/obstetrics-and-gynecology/ob-gynecology-EPAs-e-2-1.pdf
14. Lei C, Palm K. Crisis resource management training in medical Simulation. *StatPearls*. StatPearls Publishing; 2023.
15. Brown TB, Lovato LM, Parker D. Procedural sedation in the acute care setting. *Am Fam Physician*. 2005;71(1):85-90.
16. Robinson D, Basso M, Chan C, Duckitt K, Lett R. Guideline no. 431: postpartum hemorrhage and hemorrhagic shock. *J Obstet Gynaecol Can*. 2022;44(12):1293-1310.e1. https://doi.org/10.1016/j.jogc.2022.10.002
17. Neal JM, Neal EJ, Weinberg GL. American Society of Regional Anesthesia and Pain Medicine local anesthetic systemic toxicity checklist: 2020 version. *Reg Anesth Pain Med*. 2021;46(1):81-82. https://doi.org/10.1136/rapm-2020-101986
18. Panchal AR, Bartos JA, Cabañas JG, et al. Adult Basic and Advance Life Support Writing Group. Part 3: Adult basic and advanced life support: 2020 American Heart Association guidelines for cardiopulmonary resuscitation and emergency cardiovascular care. *Circulation*. 2020;142(16)(suppl 2):366-468. https://doi.org/10.1161/CIR.0000000000000916
19. Brazil V, Speirs C, Scott C, Schweitzer J, Purdy E. Recommendations for the design and delivery of visually enhanced mental simulation: insights from participants and facilitators. *J Healthc Simul*. 2025:1-11. https://doi.org/10.54531/NDXV6633

Received: August 27, 2025
Accepted: April 15, 2026
Published: June 5, 2026

Copyright © 2026 Warshafsky et al. This is an open-access publication distributed under the terms of the Creative Commons Attribution license.
